# Initial Study on COMT and DRD2 Gene Polymorphisms as Well as the Influence of Temperament and Character Trait on the Severity of Alcohol Craving in Alcohol-Dependent Patients

**DOI:** 10.3390/jcm10245892

**Published:** 2021-12-15

**Authors:** Damian Czarnecki, Marcin Ziółkowski, Jan Chodkiewicz, Anna Długosz, Joanna Feldheim, Napoleon Waszkiewicz, Agnieszka Kułak-Bejda, Marta Gorzkiewicz, Jacek Budzyński, Anna Junkiert-Czarnecka, Agnieszka Siomek-Górecka, Krzysztof Nicpoń, Aleksandra Kawala-Sterniuk, Raffaele Ferri, Mariusz Pelc, Piotr Walecki, Ewa Laskowska, Edward Jacek Gorzelańczyk

**Affiliations:** 1 Department of Preventive Nursing, Collegium Medicum, Nicolaus Copernicus University, Torun, ul. Ignacego Łukasiewicza 1, 85-821 Bydgoszcz, Poland; marcinziolkowski@cm.umk.pl (M.Z.); krzysztof.nicpon@cm.umk.pl (K.N.); 2 Institute of Psychology, Department of Clinical Psychology and Psychopathology, University of Lodz, ul. Smugowa 10/12, 91-433 Łódź, Poland; jan.chodkiewicz@now.uni.lodz.pl; 3 Faculty of Chemical Technology and Engineering, University of Science and Technology, ul. Seminaryjna 3, 85-326 Bydgoszcz, Poland; anna.dlugosz@pbs.edu.pl (A.D.); asiaf090@gmail.com (J.F.); 4 Department of Psychiatry, Medical University of Białystok, pl. Brodowicza 1, 16-070 Choroszcz, Poland; napwas@wp.pl (N.W.); agnieszka.kulak.bejda@gmail.com (A.K.-B.); 5 Department of Molecular Genetics and Justice, Collegium Medicum, Nicolaus Copernicus University, Torun, ul. Marii Skłodowskiej-Curie 9, 85-094 Bydgoszcz, Poland; gorzkiewiczmarta@cm.umk.pl; 6 Department of Vascular and Internal Diseases, Nicolaus Copernicus University, Torun, ul. Ujejskiego 75, 85-168 Bydgoszcz, Poland; jb112233@cm.umk.pl; 7 Department of Clinical Genetics, Nicolaus Copernicus University, Torun, ul. Marii Skłodowskiej-Curie 9, 85-094 Bydgoszcz, Poland; ajczarnecka@cm.umk.pl; 8 Department of Clinical Biochemistry, Collegium Medicum, Nicolaus Copernicus University, Torun, ul. Karłowicza 24, 85-092 Bydgoszcz, Poland; asiomek@cm.umk.pl; 9 Faculty of Electrical Engineering, Automatic Control and Informatics, Opole University of Technology, ul. Prószkowska 76, 45-758 Opole, Poland; m.pelc@po.edu.pl; 10 Oasi Research Institute IRCCS, Via C. Ruggero, 73, 94018 Troina, Italy; rferri@oasi.en.it; 11 School of Computing and Mathematical Sciences, University of Greenwich, London SE10 9LS, UK; 12 Department of Bioinformatics and Telemedicine, Collegium Medicum, Jagiellonian University, ul. Medyczna 7, 30-688 Krakow, Poland; piotr.walecki@gmail.com; 13 Faculty of Medicine, Collegium Medicum, Nicolaus Copernicus University, Torun, ul. Jagiellońska 15, 85-067 Bydgoszcz, Poland; ewa.laskowska@cm.umk.pl; 14 Department of Theoretical Basis of BioMedical Sciences and Medical Informatics, Collegium Medicum, Nicolaus Copernicus University, ul. Jagiellońska 15, 85-067 Bydgoszcz, Poland; medsystem@medsystem.com.pl; 15 Institute of Philosophy, Kazimierz Wielki University, ul. Ogińskiego 16, 85-092 Bydgoszcz, Poland; 16 Babinski Specialist Psychiatric Healthcare Center, Outpatient Addiction Treatment, ul. Aleksandrowska 159, 91-229 Łódź, Poland; 17 The Society for the Substitution Treatment of Addiction “Medically Assisted Recovery”, ul. Rzeźniackiego 1D, 85-791 Bydgoszcz, Poland

**Keywords:** alcohol craving, dependency, polymorphisms, genetics, cortico-subcortical loops, neurophilosphy

## Abstract

The main aim of this work was to determine the impact of COMT and DRD2 gene polymorphisms together with temperament and character traits on alcohol craving severity alcohol-dependent persons. The sample comprised of 89 men and 16 women (aged 38±7). For the sake of psychological assessment various analytic methods have been applied like the Short Alcohol Dependence Data Questionnaire (SADD), Penn Alcohol Craving Scale (PACS) or Temperament and Character Inventory (TCI) test. The SNP polymorphism of the analyzed genes was determined by Real Time PCR test. The results showed, that the COMT polymorphismmay have an indirected relationship with the intensity and changes in alcohol craving during abstinence. The DRD2 receptor gene polymorphisms are related with the intensity of alcohol craving. It seems that the character traits like “self-targeting”, including “self-acceptance”, are more closely related to the severity of alcohol craving and polymorphic changes in the DRD2 receptor than temperamental traits. Although this is a pilot study the obtained results appeared to be promising and clearly indicate the link betweengene polymorphisms alcohol craving and its severity.

## 1. Introduction

Alcoholism can nowadays be seen as a disease, and is one of the most common illnesses in the world leading to mental and physical dependence on ethanol-based drinks [1]. It is also a highly prevalent, with chronically relapsing use that, negatively affects more and more people [2].

In Poland, where all the research took place, alcohol consumption is one of the leading causes for deaths in young and middle aged people [3,4]. This is because of the high increase in alcohol consumption in the past 15 years [4]. The number of people in Poland overusing alcohol can be estimated as 2.8–3.5 million with 0.6–0.9 million of addicts. It is also said that Poland has the highest alcohol consumption rate in the world [5].

Alcohol craving is one of the symptoms of alcohol dependency and is one of the most important factors in relapse. A high intensity of alcohol craving, increasing the risk of relapse, often prolongs the alcoholism itself and makes it more complicated [6,7,8]. In accordance with the numerous researchers’ conceptions it is possible to distinguish many causes for alcohol craving that, can be summarized with several psychological and biological models:

- withdrawal symptoms [9],
- compensation [10],
- conditioning stimulus [11],
- expectation of the result [8],
- cognitive processes [12],
- three paths—expectation of reward, relief, and intrusive thoughts [13],
- sensitization of neural systems [14],
- opposite processes—disturbances in the regulation of the reward system [15,16],
- a multidimensional model of ambivalence that, recognizes the over-reactivity of the central nervous system or personality traits as the cause of hunger [17,18].

At the basis of neurobiological models, and models related to personality traits, it is possible to look for genetic changes, which may determine among the others things overactivity of the central nervous system, and, consequently, changes in behavior or emotional reactions [19].

It is important to state that there are numerous dimensions related to dependency and craving, such as among the others the: biological, psychological and environmental. The psychological domain is usually analyzed based on cognitive-behavioral aspects, and only very few studies focus on the personality traits that may play a crucial role in craving and dependency. There is a demonstrated correlation between neuroticism and introversion in alcoholism [20,21,22,23,24]. There is a strong evidence of a relationship between personality, biological factors and dependent behavior [23], though, personality is never the only predisposition to addition, as this requires combination of a number of the above mentioned factors [20,25].

One such change is the genetic polymorphism of the gene coding for the enzyme catechol-O-methyltransferase (COMT c.472G> A p.Val158Met). The COMT is an enzyme that, deactivates catecholamines, including dopamine. If the enzyme has a methionine (Met) (genotype AA), valine (Val) is up to four times less active, which causes prolongation of the half-life of catecholamines (and dopamine), particularly in the prefrontal cortex. This change can affect the function of perceptual, emotional, general activity and behavior, but also the sensation of satiety and hunger. Moreover, the literature reports that those with homozygous (AA genotype) can have better working and episodic memory, but also may have a greater predisposition to bulimic behavior [26,27,28,29].

As it has also been noted in some studies—the polymorphism in the gene encoding COMT (Val158Met) has been associated with greater susceptibility to the development of dependency [30]. Another important genetic change associated with alcohol is polymorphism of the DRD2 receptor gene encoding (c.2137G> A, p.Glu713Lys, Taq1A (rs1800497)).Thus homozygous genotype individuals AA (Lys presence) can function better cognitively [31,32]. However, the literature (see [32,33]) indicates that the DRD2 gene receptor polymorphism may also be associated with disorders being the result of taking psychoactive substances and may play a crucial role in increasing the risk of externalizing behavior.

The incidence of dependency is is explained by the presence of the DRD2 compound polymorphisms receptor gene (allele *A*) or lower density of DRD2 dopamine receptors in the striatum [34]. Simultaneous analysis of alcohol craving in relation to polymorphisms of DRD2 and COMT genes as well as temperamental and character traits (measured with the TCI scale) has been a seldom discussed research problem in alcohol -dependent persons [35,36,37].

The aim of this study was to assess the relationship between the severity of craving for alcohol and COMT polymorphisms DRD2 genes and traits of temperament and character, as well as selected clinical and anthropometric factors in patients with alcohol dependence.

The authors of this work also used Robert Cloninger’s typology, which is one of the most widely known and confirmed models of alcohol dependence. The typology of alcohol dependence according to Cloninger has a direct impact on temperament and personality traits, in this study were assessed using the TCI (Temperament and Character Inventory) scale. This model clearly shows the pathomechanisms of dependency in neurotransmission, which may be genetically determined. Similar genetic, temperamental and character changes may determine the features of alcohol dependence, including its symptoms like, craving for alcohol.

In this study we described alcohol craving as cognitive and behavioral symptoms indicating intense and uncontrollable desire to consume alcohol psychometrically evaluated by the PACS [38,39], Also, the alcohol craving can be a “strong predictor” of relapse drinking [39,40,41]. Alcohol craving is included in the criteria of alcohol dependency [39,42].

One of the models of alcohol craving is the neuroanatomical and describes the reward, inhibitory or obsessive-compulsive processes taking place in the CNS [39,43,44,45].

## 2. Material and Methods

### 2.1. Study Participants and Research Methodology

To achieve the purpose of the research we have taken the following steps: recruitment of respondents, assess of clinical (e.g., alcohol craving), anthropometric (e.g., BMI) and psychological (e.g., temperament and character traits) parameters. One of the first steps, was take of biological material to genetics analysis (a swab from a oral mucosa of respondents).

The participants’ recruitment was conducted among alcohol-dependent patients from several drug addiction treatment centers in Poland, in the following voivodeships (regions in Poland):

- Kujawsko-Pomorskie (3 centers)—males 53 (81.5%), females 12 (18.5%);
- Lodzkie (1 center)—males 11 (77.3%), females 4 (26.7%);
- Podlaskie (1 center)—males 25 (100%), females 0 (0.0%).

In total, the sample consisted of 105 men (89–84.8%) and women (16–15.2%) (aged 38±7). The patients were tested twice on the PACS questionnaire. The other tests were performed once—in the second week of treatment (hospitalization).

We selected patients in accordance with the alcohol dependence criteria as defined in the International Classification Diseases (ICD-10). The patients were studied in various clinical units (hospitals) for alcohol-dependent persons. For this purpose the SADD test was applied in order to estimate the alcohol drinking intensity.

The monthly abstinence mean time was 14.03±11.72 days and the longest last year abstinence mean time was 64.27±77.54 days. Please see Table 1.

Some drug addiction centers treat men only and additionally—the percentage of women undergoing addiction treatment is significantly lower. If one includes the risk of treatment withdrawal or participation refusal, the final percentage of women in the potential research group is very low [46,47,48].

Inclusion criteria for the study group were as listed below:

- age—at least 18 years of age,
- no other addictions but alcohol and nicotine,
- no symptoms of alcoholic and nicotine withdrawal syndrome,
- no noticeable cognitive deficits,
- no metabolic diseases (such as e.g., diabetes),
- the possibility of giving informed consent to tests, no active viral and bacterial infections, including infectious liver dysfunction.

The research was carried out using a direct interview method (face-to-face). All questionnaires and measurements were carried out by qualified professionals (experienced researchers). For this study purpose additionally 50 people (mean age 38±9), were qualified as a control group. The criterion for inclusion in the control group was:

- age—at least 18 years of age,
- no clinical diagnosis of addiction to alcohol or any other psychoactive substances (participant’s declaration),
- no symptoms of alcohol and other psychoactive substances dependence in the interview preceding the study.

Unfortunately, our experimental participants were mostly male, and the gender distribution is uneven. This is because the number of addicted male and females differ (as it has been mentioned above), however, the number of addicted women is constantly increasing [24].

The exclusion criterion from the control group was the analysis of the PACS (Physical Appearance Comparison) and SADD questionnaires.

The control group excluded those who scored more than 3 points on the PACS scale and whose answers in the SADD scale suggested problems with alcohol consumption. The control group was tested only once.

During the interview with patients and people from the control group, information was collected characterizing patients’ socio-demographic (e.g., age, marital status, place of residence, length and level of education) and clinical (e.g., length of alcohol dependence) situation using closed-type cafeteria answers.

For the study purpose the following questionnaires were used:

- Short Alcohol Dependence Data Questionnaire (SADD),
- Penn Alcohol Craving Scale (PACS),
- Temperament and Character Inventory (TCI).

All the above mentioned questionnaires (tests) are described below.

#### 2.1.1. Short Alcohol Dependence Data Questionnaire

The Short Alcohol Dependence Data Questionnaire (SADD) is a test, used to evaluate the depth of alcohol dependence [49,50]. The scale consisted of 15 questions. There were 4 variants to the given answer. Other answer variants corresponded to the degree of depth, so were scored in ascending order way from 0 to 3 points. The depth of dependence was assessed on the basis of the sum of the obtained points.

Assuming the following criteria:

- 1–9 points—mild degree of the depth of dependence,
- 10–19 points—moderate degree of the depth of dependence;
- 20–45 points—deep degree of the depth of dependence.

The applied scale provided good psychometric properties for the study, as the Cronbach’s alpha for SADD was 0.84.

The sensitivity and the specificity have not been calculated for this study purposes, however, these parameters have been known from other studies—the SADD-characterized the sensitivity is (0.80–0.81) and specificity (0.80–0.81) [51].

According to Perez-Lopez et al. [51], SADD questionnaire, regardless of the 12, 14 or 15 question version, has similar psychometric values (internal consistency between 0.91 and 0.92), which suggests that the 15-question version has similar accuracy and sensitivity in the diagnosis of the depth of addiction as the version 12-question.

#### 2.1.2. Penn Alcohol Craving Scale

For the purpose of this study the Penn Alcohol Craving Scale (PACS) was applied [52] and the authors decided to use its Polish adaptation [53]. This scale consists of five test items. Three questions were related to the frequency, intensity, and duration of craving. One measured the ability to resist temptation when drinking was possible, and another assessed the degree of overall alcohol craving in the past week. The range of given answers was from 0–6, which has good psychometric properties and is very often used in studies on alcohol craving [53,54]. In addition, it allows better prediction of the risk of relapse during therapy than other methods [55].

The range of points was as follow [53]:

- 0–3—low intensity of alcohol craving,
- 4–9—average alcohol craving,
- 10 and more—high intensity of alcohol craving.

The gender distribution in the PACS and control group was as follows:

- PACS 0–3—men: 23(88.5%), women: 3(11.5%);
- PACS 4–9—men: 36(81.8%), women: 8(18.2%);
- PACS 10—men: 30(85.7%), women: 5(14.3%);
- control group—men: 38(76.0%), women: 12(24.0%).

In the presented study, the Cronbach’s alpha for PACS was 0.82.

#### 2.1.3. Temperament and Character Inventory

The Temperament and Character Inventory (TCI) is a set of scales for measuring the dimensions of temperament and character in accordance with the psycho-biological concept of Robert Cloninger [56,57]. This method was adapted to Polish conditions by Elzbieta Hornowska in 2003 [58]. The applied questionnaire consisted of 240 questions, which, after using the key, allowed definition of temperamental areas like:

- “searching for novelty” with sub-dimensions:
  - cognitive curiosity,
  - impulsiveness,
  - extravagance,
  - disorder;
- “avoiding harm” with sub-dimensions:
  - pessimism,
  - fear of uncertainty,
  - social anxiety;
- “dependence from reward” with sub-dimensions:
  - sentimentality,
  - attachment,
  - dependence;
- “perseverance”;

and character areas:

- “self-direction” with sub-dimensions:
  - responsibility,
  - purposefulness,
  - resourcefulness,
  - self-acceptance,
  - good habits;
- “willingness to cooperate” with sub-dimensions:
  - social acceptance/tolerance,
  - empathy,
  - willingness to help,
  - forbearance,
  - integrated conscience;
- “ability to detach from oneself/transcendent self” with sub-dimensions:
  - creative self-transgression,
  - trans-personal identification,
  - acceptance of spirituality.

Each of the dimensions and sub-dimensions was summarized with a calculation algorithm. The results of individual dimensions and sub-dimensions in this study were compared with the results of the standardization group and interpreted by clinical psychologist [58]. The study of temperamental and character traits does not apply to the control group.

### 2.2. Conducted Anthropometric and Aenetic Analyses

For the purpose of this study the following anthropometric and aenetic analyses were performed:

- nutritional assessment by anthropometric method,
- gene polymorphisms designations.

#### 2.2.1. Nutritional Assessment by Anthropometric Method

For the purpose of this study TANITA MC-780 S MA multi-frequency segment body composition analyzer was used to carry out nutritional assessment. The conducted examination was non-invasive as it was based on the electrical bioimpedance method (BIA) [59]. The device is medically certified and can be used for scientific research. Using the above mentioned device it was possible to determine the following:

- adipose tissue content, i.e., fat mass (Fat Mass, FM, kg),
- percentage of body fat (% Fat Mass),
- content of lean body mass (Fat Free Mass, FFM, kg),
- Body Mass Index (BMI, kg/m${}^{2}$) based on body weight (kg) and stature (cm),
- metabolic age (years),
- phase angle to assess the overall participant’s health.

#### 2.2.2. Gene Polymorphisms Designations

Isolation of the DNA from oral mucosa swabs was performed with the use of professional, disposable swabs. Sampling took place approximately 2 h after a meal or on an empty stomach. Moreover, the study participants were not allowed to smoke cigarettes or chew gum for about 2 h after the study.

The SNP (Single Nucleotide Polymorphisms) polymorphism of the analyzed genes was determined by a Real Time PCR (Polymerase Chain Reaction) test. The genotyping was performed using commercial Taqman Genotyping Master Mix kits and TaqMan SNP Genotyping Assay in accordance with the manufacturer’s instructions on a ViiA7 (Applied Biosystems) apparatus (see details in Table 2). with the addition of 2.5 ng of template DNA to the reaction. The analysis of the obtained results was performed using Ruo ViiA 7 Software. The presence of the different types of polymorphic variants was confirmed by direct sequencing using the BigDye Terminator v3.1 Cycle Sequencing Kit (Applied Biosystems) also according to the manufacturer’s instructions. Sequencing products were separated in POP7 polymer on an ABI3130 apparatus. The analysis of the results was carried out with the use of SeqScape v.2.5 software.

The population of patients and control group is at Hardy Weinberg equilibrium, calculated with the Formula (1):

(1) $${\left(p+q\right)}^{2}=p^{2}+2pq+q^{2},$$

where:

*p* is the frequency of the dominant gene,

*q* is the frequency of the recessive gene.

To assess the population balance, we decided to use a a software for Microsoft Windows, which is available free of charge from and was described in: [60].

**Figure 1:**
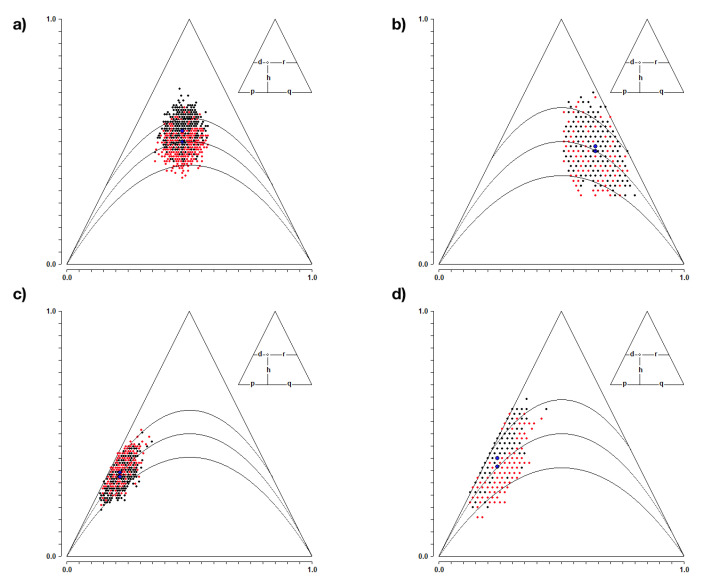
Population balance analysis according to Hardy-Weinberg, where (**a**) patients with variants of the COMT gene, (**b**) control group with variants of the COMT gene, (**c**) patients with variants of the DRD2 receptor gene and (**d**) control group with variants of the DRD2 gene.

As seen in Figure 1 the population balance analysis showed no differences in the frequency of heterozygous alleles and homozygous polymorphisms of the DRD2 and COMT receptor genes as well in patients as in the control group. Thus, the null hypothesis was not rejected, which was tantamount to adopting the Hardy-Weinberg population equilibrium.

Based on the isolated DNA, the following gene polymorphisms were determined:

- Val158Met of the COMT gene—a marker of the depressive reaction in the face of stress and also of impulsivity,
- Taq1A of the DRD2 gene—marker of increased alcohol consumption and also of impulsivity.

### 2.3. Analysis and Statistical Methods

The compliance with the normal distribution was checked for the analyzed variables. The Shapiro-Wilk test was applied in order to assess the distribution, which showed that the analyzed variables deviated from the normal distribution. This resulted in using the non-parametric U Mann-Whitney, Wilcoxon, Kruskal-Wallis, linear regression test being used for the purpose of statistical analyses. The Pearson Chi-square test with Yates’ correction was used for the comparison of the variables frequency. During this study tests from the IMAGO6 statistical package (IBM SPSS 26) were implemented. The authors of this work decided to use the mean (*X*) and statistical deviation (SD) of the obtained data in the presented statistical descriptions. The level of statistical significance was p≤0.5.

### 2.4. Funding and Ethics

This study was funded by the National Health Program: “Supporting research in the area of risk factors and factors protecting against problems resulting from alcohol consumption” (grant no.: 74/44/3.4.3/18/DEA).

Ethical approval was obtained from the Bioethics Committee (consent no.: KB692/2017).

## 3. Results

Table 3 presented both sociodemographic and clinical variables obtained from patients, hospitalized for alcohol dependency and those from the control group.

### Mann-Whitney U Test/Chi-Square

The experimental and control groups differed in terms of the length of education (which was longer in the control group) and marital status (greater percentage of divorced and single people in the experimental group).

In Table 4 presents a comparison for the COMT polymorphisms in the subpopulations of patients separated in accordance with their severity of alcohol craving (PACS scale) and the control group.

In the compared subgroups of patients and the control group (see Table 4), no differences were found between the percentages of people with polymorphisms—heterozygote G/A. It has been shown that the highest percentage without the COMT polymorphism is in the group of patients with strong alcohol craving compared to the control group. A significantly lower percentage of polymorphism—mutational homozygote A/A compared to the control group was demonstrated in patients with an average alcohol craving value.

Table 5 presents a comparison of the frequency of polymorphisms in the DRD2 receptor gene in subpopulations of patients in accordance with their severity of alcohol craving (according to the PACS results) and the control group.

In the compared patient subgroups and the control group (see Table 5), no differences were found between the percentages of people without DRD2 polymorphism. The lowest percentage of polymorphisms—the heterozygote G/A was demonstrated in patients with low alcohol craving compared to patients with moderate and high alcohol craving values as well as to the control group. A statistically significantly higher percentage of Polymorphism—mutational homozygote A/A (of clinical significance) was demonstrated in patients with low alcohol craving compared to patients with strong alcohol craving (15.4 vs. 0%).

Comparison of mean PACS values between patient subpopulations according to the presence or absence of the COMT and DRD2 polymorphism showed a difference in the severity of alcohol craving depending on the DRD2 polymorphism (Table 6).

**Figure 2:**
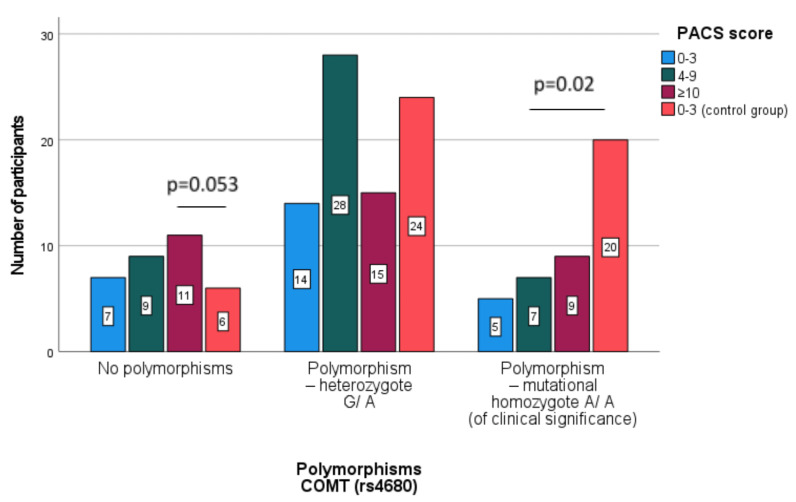
Comparison of the subpopulations participants separated according to the severity of alcohol craving (PACS scale) in relation to the COMT polymorphisms (Pearson Chi-square with Yates’ correction).

**Figure 3:**
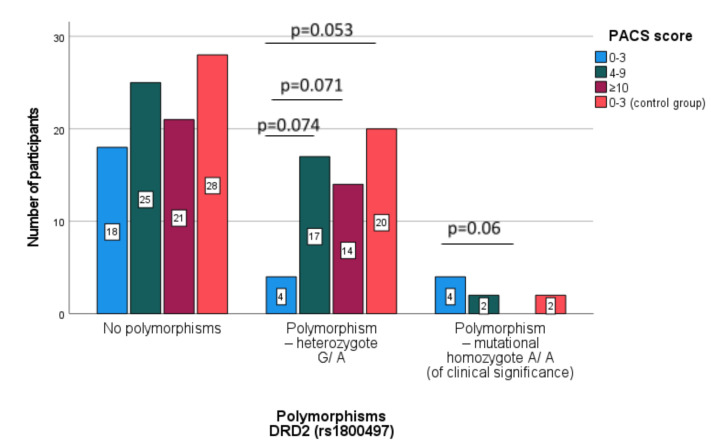
Comparison of the subpopulations participants separated according to the severity of alcohol craving (PACS scale) related to the DRD2 polymorphisms (Pearson Chi-square with Yates’ correction).

In Figure 2 comparison of the subpopulations participants separated in accordance with the alcohol craving severity in relation to the COMT polymorphisms was presented, where Figure 3 illustrates the same participants but in relation to DRD2.

**Figure 4:**
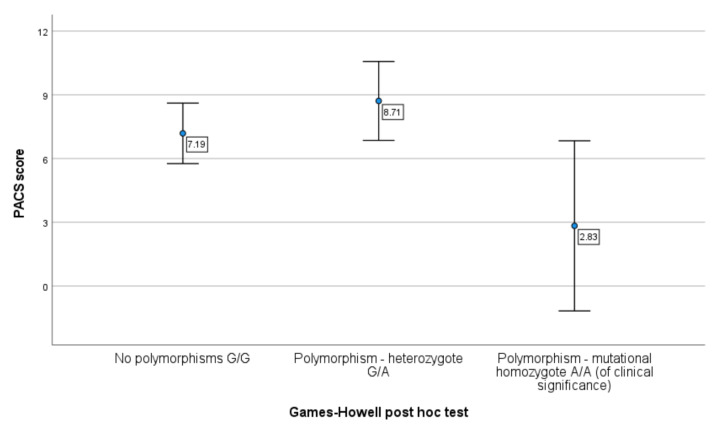
Comparison of the PACS score (severity of alcohol craving) according to polymorphisms in the DRD2 receptor gene in subpopulations of patients with alcohol dependence.

Figure 4 shows the difference in the severity of alcohol craving depending on the presence or absence of DRD2 polymorphism. Patients with polymorphism—mutational homozygote A/A (DRD2) were characterized with lower alcohol craving than patients with the DRD2 polymorphism—heterozygote G/A (p=0.025) and no polymorphism G/G (p=0.087).

**Figure 5:**
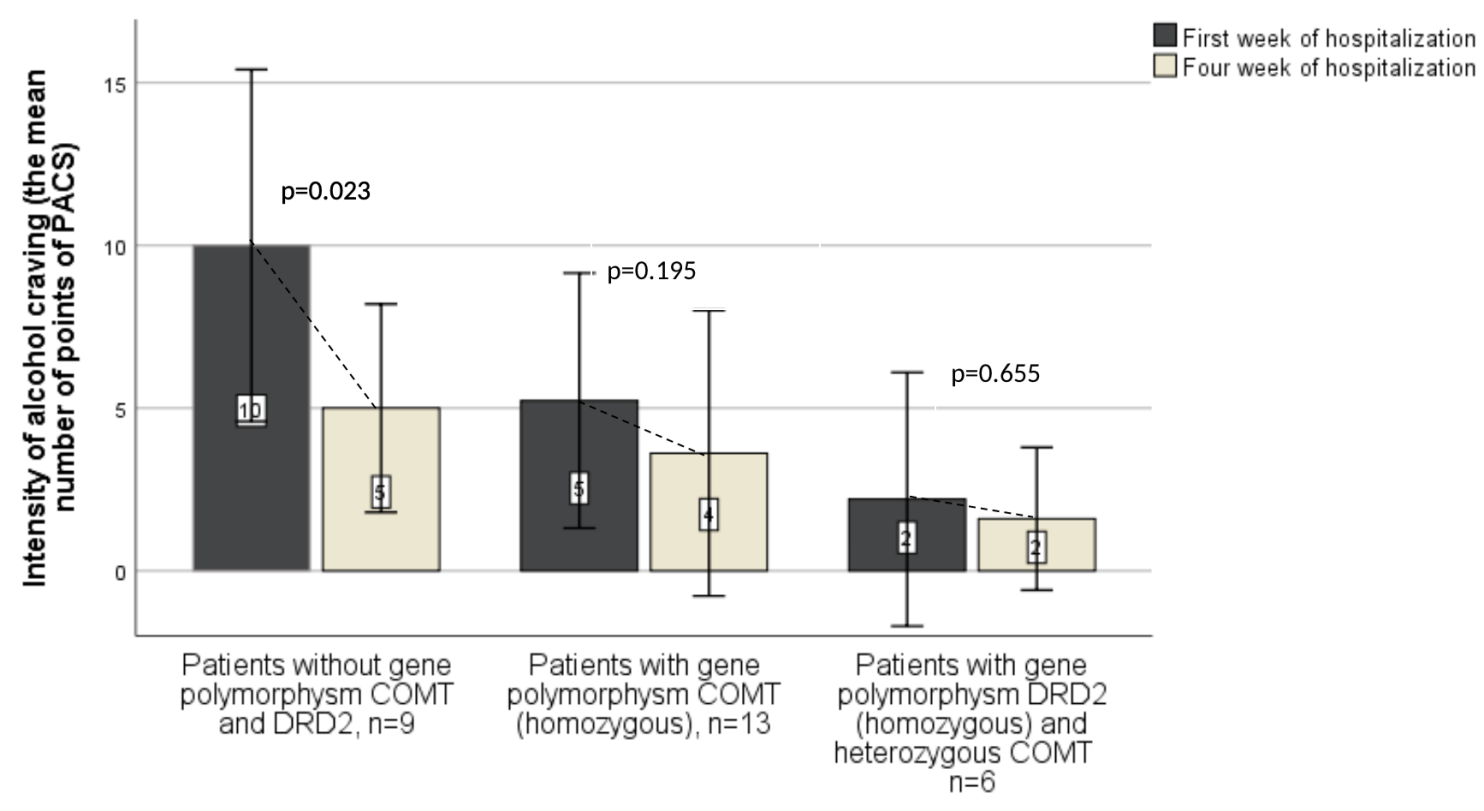
Changes in the intensity of alcohol craving (PACS scores) in groups of patients with or without polymorphisms in the COMT and DRD2 genes over a 4-week period, counting from the second to the sixth week of hospitalization (Wilcoxon test).

In Figure 5 changes in the intensity of alcohol craving, based on the obtained PACS scores, in groups of patients with or without polymorphisms in COMT and DRD2 genes over a 4–week long period, counting from the second to the sixth week of hospitalization (Wilcoxon test), were illustrated. The authors sought to extract the most numerous subpopulation of patients with just the DRD2 polymorphism (homozygote) as changes of clinical significance, without the presence of other DRD2 polymorphisms (heterozygotes) and the COMT polymorphisms. However, this was challenging so, it was decided to leave the connection in four out of six patients—the DRD2 homozygotes and the COMT heterozygotes.

To the linear regression model were included variables in which, based on previous analyzes, the differences in the subpopulation of patients separated according to the severity of alcohol craving (see Table 6, Table 7 and Table 8) were shown: DRD2 polymorphism, and TCI subscales—”Award dependence, Affection, Perseverance, Self-Direction, Self-acceptance, Willingness to cooperate, Empathy, Readiness to help”.

Table 7 and Table 8 present clinical characteristics together with temperamental and character traits of the sub-population of patients experiencing different levels of alcohol craving.

Comparison of clinical variables, temperament and character between the subpopulations of patients distinguished according to the severity of alcohol craving and the control group showed no differences in the average values of age, length of abstinence (in months), body weight, BMI, phase angle, temperament traits—curiosity, insecurity, social anxiety, dependency, and social acceptance/tolerance, indulgence, spirituality character traits (see Table 7 and Table 8).

Most clinical and anthropometric variables did not differ depending on the severity of alcohol craving patients’ groups. It was shown that the temperament traits “Award dependence” and “Perseverance” were significantly more pronounced in the group with low than moderate alcohol cravings. On the other hand, the temperament trait “Affection” was significantly less expressed in the medium rather than high intensity of alcohol craving character group.

In the linear regression model (Table 9), variables of temperament and character as well as DRD2 polymorphism remained. It was shown that the “Self-acceptance” character trait was significantly related to the intensity of alcohol craving (PACS) as was “Polymorphims DRD2” and the temperament trait “Perseverance” at statistical significance. The entire model obtained an average level of $R^{2}=0.211$, which means that it explains only 21% of the variance with other factors responsible for the remaining, which should be investigated in the future.

Character traits like “Self-Direction”, “Self-acceptance”, “Willingness to cooperation”, “Empathy”, “Readiness to help” were more apparent in the low rather than average alcohol craving group. In addition, the characteristics of “Self-Direction” and “Self-acceptance” were also more expressed in the high intensity of alcohol craving group.

Comparison in terms of clinical and anthropometric variables as well as TCI results of the control group, which was characterized with the low alcohol craving (PACS 0–3 score), and the other patient subgroups, reveled that:

- subjects in the control group differed significantly from all subgroups of patients with varying degrees of hunger: length of dependence, standard drinks over the month, the longest time of abstinence on the last year (days), SADD and “Search for novelty” (trait weakly expressed), “Extravagance” (trait weakly expressed), “Fatigue and asthenia” temperament traits and “Responsibility” (trait more expressed), “Self-transcendence” (trait weakly expressed) character traits. The variables seem to be independent to alcohol craving intensity;
- subjects from the control group were characterized by significantly lower severity of pre-meal hunger, and less pronounced “Avoiding harm”, “Pessimism” temperament traits compared to the group of patients with strong alcohol craving;
- patients in the control group had more fat tissue (% FM) than patients with low and moderate alcohol cravings;
- patients from the control group were characterized by a less pronounced “Impulsiveness” temperamental trait and more pronounced features of “Affection”, “Resourcefulness”, “Willingness to cooperation”, “Readiness to help” than patients with moderate craving for alcohol;
- subjects from the control group were characterized by less expressed temperamental trait “Avoiding harm” temperamental trait and more pronounced character traits like “Self-Direction”, “Desirability of proceeding”, “Self-acceptance”, “Second nature” than patients with average and high values alcohol craving;
- patients from the control group were characterized by a less pronounced “Sentimentality” temperamental traits than patients with low alcohol craving.

## 4. Discussion

The performed study sough to identify the relationship factors determining the severity of alcohol craving, such as polymorphisms of genes and traits of character and temperament. This topic covered a small but important area in understanding the factors, that, either prevent or increase the risk of relapse into alcohol consumption [61].

The role of cortico-subcortical loops in dependence was postulated [62]. The potential role of distinct genetic influence on the structures of the brain was indicated. Studies of cue-elicited craving report activation in brain regions that constitute dopamine pathways. Also, increases in cerebral blood flow in structures like the amygdala and anterior cingulate—regions of the limbic system of cocaine users while watching drug use videos were reported in literature [63]. The dopamine-rich regions such as the ventral tegmental area (VTA) were implicated in the neuroplasticity concerning the development of dependence and the emergence of craving [64]. The genes in the dopamine pathway may yield promising insights regarding the genetic contributions to craving. Alcoholic craving may result from overactivity within the fronto-thalamic neuronal loop, and loss of control of alcohol consumption resulting from impairment of the limbic—striatal circuit caused by the dopaminergic effects of intoxication [62]. The striatal involvement in human alcoholism and alcohol consumption, and withdrawal was postulated also in animal models [62].

It is possible that either, the presence or absence of individual DRD2 and COMT polymorphisms are associated with the severity of alcohol craving. Table 7 and Table 8 show that the COMT A/A polymorphism is not significantly related to the strength of alcohol craving, because in the group of patients with the PACS > 10 points, the frequency of this polymorphism was 25% and in the control group with the PACS 0–3 points 40%, there was no statistically significant difference between these frequencies, unlike in the case of DRD2 A/A polymorphism, which seems to be related to the severity of alcohol craving because there is a significant difference in the frequency of this polymorphism, the group of patients with PACS 0–3 points and the group of patients with PACS > 10 points (15% vs. 0%), i.e., the presence of DRD2 A/A polymorphism may be protective with regards to the intensity of alcohol craving.

This study also tried to reveal to what extent either, presence or absence of polymorphisms in alcohol dependent patients is related to changes in alcohol craving during abstinence. The graph (Figure 5) shows that people without COMT and DRD2 polymorphisms had high levels of alcohol craving, which statistically significantly reduced in intensity during 4 weeks of abstinence. The situation is different in subjects with COMT (A/A) polymorphism, who had half the severity of craving for alcohol compared to patients without COMT/DRD2 polymorphisms at the beginning of hospitalization, and the severity of craving did not show a statistically significant decrease during the 4 weeks of abstinence. The same applies to patients with DRD2 (A/A) polymorphism. However, it is worth adding that the severity of alcohol craving was even lower at the beginning of the therapy and remained at a similar level over 4 weeks of abstinence/hospitalization.

Knowing the frequencies of polymorphisms (especially A/A polymorphisms) in groups of patients with increased alcohol craving PACS with scores: 0–3, 4–9 and >10, it is possible to determine what temperament and personality traits can be observed in particular sub-populations.

Taking into consideration the COMT polymorphism and its form—the A/A homozygous mutation, which shows the most confirmed clinical significance, the authors did not detect a direct relationship with the intensification of alcohol craving (exactly with the results from the PACS scale), but rather with the course of alcohol dependence itself. This interpretation is related to the presence of a similar percentage of the COMT A/A mutation in patients from the subpopulation with low, medium and high alcohol craving, and the only differences in the frequency of this polymorphism are between the control group, which can be characterized with low alcohol craving, and the subpopulation of patients with medium alcohol craving. The average intensity of alcohol craving was 40% and 16% of the A/A mutation, respectively. However, Blum et al., have shown in their studies (see [65] that, inter alia, inhibiting COMT may be useful in reducing alcohol craving and thus the risk of relapse.

It is possible that the above suggestion reflects analyzes presented in this paper illustrated with the Figure 5.

In case of the frequency of DRD2 receptor polymorphism, a certain gradation relationship can be observed with the intensity of alcohol craving. The frequency of the DRD2 polymorphism, in the form of the A/A mutation homozygous, was higher in the subpopulation of patients with low alcohol craving that with high (15% vs. 0%—respectively). At the same time, no statistically significant difference was found between the frequency of DRD2 receptor polymorphism (A/A mutation homozygous) in patients with high levels of alcohol craving and the control group, which experienced this craving only insignificantly. According to other researchers’ reports, this is correct concept, because a lower sensitivity of dopaminergic receptors (DRD2) in the nucleus accumbens should contribute to an increase in the feeling of hunger, and increasing this sensitivity lead to its reduction [65]. Studies published in 2007 indicated a relationship between the reduction of DRD2 receptors and a higher risk of alcohol consumption and alcohol craving [66].

Further analysis of the relationship between COMT and DRD2 gene polymorphisms and the severity of alcohol craving showed that patients who did not have any form of COMT and DRD2 polymorphism revealed significantly increased craving for alcohol at the beginning of drug dependence treatment, and its intensity decreased statistically during monthly abstinence. In contrast, patients with only the COMT (A/A) mutation (without the DRD2 receptor mutation) had significantly lower craving for alcohol, which remained at a similar level for 4 weeks of abstinence (with a downward trend). In turn, in patients with the DRD2 receptor mutation (it was difficult to distinguish a pure group only with the A/A mutation of the DRD2 receptor gene), the intensity of alcohol craving at the beginning of therapy was very low and remained at a similar level during continued abstinence.

The analysis of the relationship between the severity of alcohol craving, COMT and DRD2 polymorphisms and temperament and character traits showed that in patients with low alcohol craving (at the same time, it is known from earlier analyzes that with a higher frequency of A/A polymorphism of the DRD2 receptor), the TCI subscales as traits were higher for temperament (such as “Reward dependency”, “Perseverance”) than patients with average alcohol craving. In terms offore urobiology, this feature is conditioned more by the functioning of the noradrenergic system than the dopaminergic system [56,57,58].

However, it is known that the COMT met allele is associated with a reduction in the COMT, and this in turn is linked to greater extrasynaptic dopamine accumulation and greater sensitivity of the dopamine receptor in people pathologically overconsuming alcohol [67].

Unfortunately, it is difficult to explain the phenomenon of differences in the values of the TCI subscales between the patients with low intensity of craving and average craving for alcohol with, no such differences between the patients with low intensity and strong craving for alcohol.

Due to the variations in the DRD2 (A/A) polymorphism differences between patients with low and high alcohol craving, the assessment should also include differences in the TCI scores only for the “self-targeting” traits, including the “self-acceptance” personality traits sub-dimensions, which were more expressed in patients with low craving than in those with high intensity of alcohol craving [56,57,58,68].

Other studies indicated the above mentioned relationship, as it was shown that the relationship of the DRD2 receptor with the actions directed at the target. Moreover, increased expression of the DRD2 receptors minimizes alcohol consumption [66,69].

People with high values of the “self-direction” sub-scale are considered to be mature, constructive, responsible, independent, easily and consciously adjusting their behavior to their goals. On the other hand, those with low scores can be characterized as of “weak structure” who, act ineffectively. They are perceived as immature, with a certain internal disorganization that makes it impossible to set and achieve goals [56,57,58]. In a general sense, the driving force behind their activity are external stimuli rather than internally determine motives [56,58]. The sub-dimension of “self-direction” is “self-acceptance”, the opposite of which is “struggling with yourself” [56,57,58]. People with high values of the self-directed dimension are characterized by self-confidence and acceptance of their good and bad sides. Conversely, people with low values of this sub-dimension do not accept their characteristics (physical and mental) [58].

The above-mentioned positive qualities (constructive, deliberate action, self-confidence and self-acceptance) may contribute, for example, to better coping with stress, and this in turn reduces the craving for alcohol. Also, traits such as careless and ill-considered actions and lack of planning may lead to increased craving for alcohol and, consequently, to relapse [70].

It is worth mentioning that this study shows how the sub-populations of patientsselected according to the severity of alcohol craving differ in the results of the TCI subscale “novelty seeking”, and the severity of alcohol dependence (SADD scores) [71].

### Study Limitations

Research limitations include the low number of patient subpopulations identified through the data analysis process.

The study has analyzed only the COMT and DRD2 polymorphisms, which the authors are going to expand on in their further studies with additional experiments on polymorphisms of the 5—*HTTLPR* (serotonin-transporter-linked polymorphic) gene for a full neurobiological reference to the Cloninger alcohol dependence typology and the TCI temperament and character questionnaire, such as inter alia [69,72].

It was possible to sequence the entire genome (NGS) (Next-Generation Sequence), but the task exceeded the financial possibilities of the grant. Therefore, some of the best known gene polymorphisms in the field of dependence have been selected though, but we are aware there are many other genetic changes that affect the process of alcohol dependence.

Another significant limitation of this study is gender inequality in both dependent patients and control group. This is because the study has been carried out in Poland and here most of the dependency treatment centers treat men only and most studies focus on male treatments and dependence only [46]. This is because historically—drug and alcohol dependence were considered to affect men only [48,73]. The global statistics state that men consume more alcohol than women, as 54% of males (1.46 billion) and only 32% of females (0.88 billion) aged 15+ consumed alcohol [47]. Gender inequality is a large problem in dependence related studies [20,24].

The percentage of women in the study was very low, because of the difficulty in gaining access to centers where only women dependence was dealt with. There are gender-related differences in inter alia neuroticism or impulsiveness, which are important personality factors, therefore investigations with these factors being taken into consideration should provide interesting results. It would be also useful in the future to analyze how these personality traits affect craving in women and their vulnerability to alcohol dependence. Another important factor is age, as it is claimed that personality itself is less stable and not fully developed before the age of 30 [20,24].

Some studies also pointed out that there is a difference on impact of mood on men and women, in particular for alcohol craving [24].

Temperamental traits are in mutual relationship with character traits [56,57]. In this work the authors demonstrated the relationship of the character traits of “self-directedness”, including its sub-dimension of “self-acceptance” with the severity of alcohol craving. A relationship, as yet not entirely clear, between the increase of alcohol craving and the temperamental trait “reward dependence” has been noticed. This trait, as discussed by the numerous researchers, is conditioned by the dopaminergic system, which may also promote the development of personality traits like self-direction and/or self-acceptance (high values of self-confidence and acceptance dimension, greater goal-oriented motivation) [56,70,74], that, as it was shown, is related with the lesser experience for alcohol craving.

In terms of neurobiology, the studied parameters like, receptor gene polymorphisms (DRD2), have an impact on the functioning of the dopaminergic system in the brain structures, which directly or indirectly affect the development of personality traits, stimulating and maintaining motivation for action [75]. The DRD2 receptors are present in high density in the striatum (nucleus accumbens) as postsynaptic receptors, and in the ventral tegmental and substantia nigra fields as autoreceptors, with moderate density in the frontal lobe [76]. The DRD2 receptors are coupled with the Gi protein, and are an inhibitor of adenylate cyclase, which reduces the synthesis of cAMP. As autoreceptors, they affect the synthesis of dopamine, and as presynaptic receptors, in e.g., in the cortico-striatal tract, they affect the concentration of glutamate [77].

A 2011 study (see [75]) showed a relationship between the DRD2 polymorphism (rs 1800497) and the development of alexithymia, which is associated with difficulties in awareness and self-regulation of emotions [75,78,79] and it seems that this trait could be related to the personality trait studied by us, which is “self-directed, including self-acceptance”. People with alexithymia have less activation of “anterior cingulate cortex (ACC)”, and thus process emotional stimuli differently [78,80,81,82].

Other studies indicate the simultaneous presence of factors predisposing to other dependence such as inter alia heroin or cannabis dependence, including DRD2 polymorphism (rs 1800497, homzygotes) and personality traits like neuroticism, extraversion ans/or introversion, psychoticism) [81,83,84,85,86]. In addition, researchers have shown that there is a relationship between changes in the functioning of the dopaminergic system conditioned by asymmetric dopamine signaling in the striatum and frontal regions, and the involvement of DRD2 receptors (rs 1800497) in these changes, which ultimately affects cognitive processes. In the above-mentioned studies, no relationship between the COMT Val158Met polymorphism and the analyzed processes was demonstrated [87].

The reward system regulates the motivation conditioned by emotional processes in appetitive behavior by—strengthening or reducing the pursuit of goals. Similarly, the prefrontal cortex plays a role in building motivation, but also in planning and organizing behavior [88,89]. It is also worth mentioning that many studies confirm the role of the reward system (including the nucleus accumbens) in the development of dependence, but also in the search for psychoactive substances and the feeling of wanting to take them [90,91,92].

To summarize it is generally recognized that the brain centers like the reward system or the frontal cortex, in which the dopaminergic system plays a large role, control and modulate emotions, behavior and learning processes, which may also shape temperamental and personality traits and ultimately affect the degree of coping with abstinence.

The results of this study research showed that the risk of relapse drinking alcohol expressed by the PACS score (>10 score) may be particularly high in patients without DRD2 polymorphism G/G and with heterozygous DRD2 polymorphism G/A. On the other hand, the presence of homozygous DRD2 A/A polymorphism seems to be protective, i.e. minimizing the risk of relapse into drinking alcohol, as patients with this polymorphism experienced an average alcohol craving (<3 score) (Figure 4).

It can be assumed (see Figure 5) that the highest risk of relapse into alcohol consumption (the highest PACS values) occurs at the beginning of therapy in patients with alcohol use disorders without the COMT and DRD2 polymorphisms. Additionally, we obtained interesting observations regarding the coexistence of three factors associated with the high PACS values, and thus an increased risk of recurrence. These factors include the low expression of the temperament trait “perseverance” and the character trait “self-acceptance” as well as the lack of polymorphism of the DRD2 receptor gene.

## 5. Conclusions

Based on the achieved results, the following conclusions can be drown:

- the COMT polymorphism may have not-necessarily direct relationship with the intensity and changes in alcohol craving during abstinence;
- DRD2 receptor gene polymorphisms are related with the intensity of alcohol craving;
- it seems that the character traits such as “self-targeting”, including “self-acceptance”, are more closely related to the severity of alcohol craving and polymorphic changes in the DRD2 receptor than temperamental traits.

We observed that the homozygous DRD2 A/A polymorphism was associated with less alcohol craving. The linear regression model (see Table 9) showed that in approximately 20% of the variance, alcohol craving is related to the coexistence of several factors such as A/A polymorphism, “Perseverance” and “Self-acceptance” features while, the remaining 80% depends on other factors that, should be looked into in further research.

The regression model indicated that there is a negative relationship between the above variables and craving for alcohol, which was confirmed by further analyzes (Table 7 and Table 8). It should be clarified that in the regression model the homozygous DRD2 A/A polymorphism was associated with a higher expression of the Perseverance temperamental traits and the self-acceptance character, and also when the homozygous DRD2 A/A polymorphism was present, the patient experienced less alcohol craving. More pronounced “Self-acceptance” and “Perseverance” features were observed in the patients’ group and the control group with less than higher levels of alcohol craving.

Additionally, we have shown (Table 5) that there was a higher frequency of homozygous DRD2 A/A polymorphism in patients with PACS 0–3 scores than with PACS >10.

The analysis of differences between the control group and the subpopulations of patients distinguished according to the severity of alcohol craving, suggested that pre-meal hunger, the percentage of body fat and features such as “Search for novelty”, “Fatique and asthenia”, “Responsibility”, “Self- transcendence”, “Extravagance”, “Pessimism” and, “Avoiding harm” were more related to dependence itself than to alcohol craving (except for ““Responsibility”, the other mentioned variables were less expressed in the control group than in patients). This suggestion is confirmed by the differences in the variables between the control group (PACS 0–3) and all subpopulations of patients distinguished according to the severity of alcohol craving, or those subpopulations of patients with low (PACS 0–3) or moderate (PACS 4–9) intensity of alcohol craving. On the other hand, features such as “Self-direction”, “Desirability of proceeding”, “Self-acceptance”, “Second nature” were more expressed in the control group than in patients with moderate and high intensity of alcohol craving. Hence, it can be presumed that the relationship between the above features is greater with alcohol craving than with the other variables differentiating the control group and patient subpopulations. The above results may be useful in assessing the risk of relapse into alcohol consumption and the selection of therapeutic approaches in working with and coping with alcohol craving.

The study of Vitali et al., showed that the alcohol craving intensity and frequency was correlated with “Harm Avoidance” (TCI), and not with “Novelty seeking” (TCI) [39,93]. Vitali et al., also showed that “Self Directedness” was negatively correlated with intensity and frequency alcohol craving. The positive traits “Novelty seeking” and negative “Cooperativeness” were correlated with the severity of alcohol dependence [39].

Evaluation of alcohol craving in the context of character or temperament traits, neurotransmiters tract and genetics is very important because of the connection to a more personalized analysis of alcohol-dependent patients. Some researchers are even in the process of using a pre-screening tool (Genetic Addiction Risk Score (GARS)) [39], where it can be seen that a decrease in “Self-directedness” was connected with the severity of alcohol problems, higher frequency of family and social problems, employment and support problems or psychiatric problems. Additionally, a strongly expressed “Self-transcendence” trait was correlated with more frequent medical problems. Their study confirmed our results, because we have seen that more characteristic for the control than the patients’ group were traits like “Self-directedness” for example high SADD, fatigue and asthenia. The “Self-transcendence” trait was more characteristic for patients.

The use of the Cloninger inventory in many studies indicates an ambiguous relation between individual character traits and temperaments with alcohol dependence. Some researchers indicate that in dependent patients “Avoidance of Damage”, “Search for Novelty” and low values of the “Self-Direction” are particularly marked, which is consistent with our results [94,95,96].

The Alvarez et al. [96] study showed that alcohol craving (OCDS score) was connected with “Search for novelty” (positive correlation), “Avoidance of Damage” (positive correlation), “Persistence” (negative correlation), “Self-direction” (negative correlation), “Cooperation” (negative correlation). This means that for example, persons who are ambitious (“Persistence”), are, more creative (“Self-transcendence”). These results were similar to our outcomes, except for “Self-transcendence“, which was statistically less significant in the control group than in patients. Similar results were obtained by Tavares et al. [97], in which it was pointed out that alcohol dependent are characterized, among others, by Novelty seeking as an indicator of greater susceptibility to anxiety. In addition, the same authors noticed that people with anxiety, “Avoiding of damage” and alcohol craving particularly often consume alcohol. This means that they consume alcohol in the face of negative emotions.

The results of our research are consistent with the suggestions of other authors which discuss possibilities of e.g., Genetic Addiction Risk Score application [39]. Our studies and that of other authors’ have shown that in predicting the risk of relapse to drinking (promoted by the strength and frequency of alcohol craving), we can use the so-called “genetic-temperamental-characterological” diagnosis, which increases the chance of identifying “patient characteristics”. This suggests the implementation of specific and targeted forms of therapy (support, education, psychotherapy and, pharmacotherapy). Getting to know these “traits” allows for their modulation (reduction, promotion), e.g., with worse values of the traits “Self-direction”, “Self-acceptance”, “Perseverance” (education, cognitive-behavioral psychotherapy), and with “Pessimism” (support, antidepressants) [98,99]. What is special in the individual selection of diagnostic and therapeutic methods is the determination of the coexistence of positive (e.g., enhancement of the “Self-direction”, “Self-acceptance”) or negative features (e.g., enhancement of “Pessimism” and weakening of the “Self-direction” feature) with the presence (protection of hunger) or the absence (lack of protection of hunger) of the homozygous DRD2 polymorphism. The conjunction of “negative traits” will take a great deal of effort on our part to help patients maintain abstinence and, reducing the occurrence of alcohol cravings. On the other hand, the conjunction of “positive qualities” will be an ally in standard abstinence activities.

## Figures and Tables

**Table 1:** Mean time of abstinence.

	Patients, n=105
	Mean ± SD
Time of abstinence on the month (days)	14.03±11.72
The longest time of abstinence on the last year (days)	64.27±77.54

**Table 2:** Genotyping of polymorphisms—SNPs and TaqMan.

SNP Polymorphisms	TaqMan SNP Genotyping Assay
gene COMT (rs4680)	C__25746809_50
gene DRD2 (rs1800497)	C__7486676_10

**Table 3:** Sociodemographic and clinical variables in patients hospitalized for alcohol dependency and the control group.

Variables	Hospitalized Patients *N* = 105	Control Group *N* = 50	*p*
Age (years)	38.1±7.0	38.3±9.8	0.841
Gender:			
females	16 (15.2%)	12 (24.0%)	0.185
males	89 (84.8%)	38 (76.0%)	
Education duration (years)	11.61±2.93	16.56±1.78	0.0005
Place of residence:			0.615
rural	27 (25.7%)	11 (22.0%)	
urban	78 (74.3%)	39 (78.0%)	
Marital status:			0.001
married	34 (32.4%)	34 (68.0%)	
in civil partnership	10 (9.5%)	5 (10.0%)	
divorced	18 (17.1%)	2 (4.0%)	
widowed	2 (1.9%)	0 (0.0%)	
single	40 (38.1%)	9 (18.0%)	
no data	1 (1.0%)	0 (0.0%)	
Duration of dependence (years):	12.46±7.04	0.00±0.00	0.0005
PACS (points)	7.45±5.63	0.30±0.76	0.0005
SADD (points)	23.71±6.95	0.58±1.14	0.0005
Height (cm)	175.46±8.42	178.78±8.85	0.030
Weight (kg)	78.48±15.77	83.57±19.91	0.147
BMI	25.38±4.25	25.88±4.86	0.479
FM %	20.26±6.80	24.20±7.35	0.001
General condition			
Phase angle	5.98±0.80	6.03±0.62	0.874
Metabolic age (years)	33.70±12.21	38.93±12.42	0.006

**Table 4:** Comparison of the frequency of COMT polymorphisms in the patients subpopulations separated according to the severity of alcohol craving (PACS scale) and the control group.

	Study			Control
	Participants			Group
	**A**	**B**	**C**	**D**
Gene	Patients	Patients	Patients	Subjects
polymorphisms	with PACS	with PACS	with PACS	with PACS
COMT (rs4680)	0–3 points	4–9 points	>10 points	0–3 points
	N=26	N=44	N=35	N=50
No polymorphisms;	7 (26.9%)	9 (20.4%)	11 (31.4%) ${}^{1}$	6 (12.0%)
G/G				
Polymorphism—	14 (53.8%)	28 (63.6%)	15 (42.9%)	24 (48.0%)
–heterozygote				
G/A				
Polymorphism—	5 (19.3%)	7 (16.0%) ${}^{2}$	9 (25.7%)	20 (40.0%)
–mutational				
homozygote A/A				
(of clinical significance)				

Pearson Chi-square with Yates’ correction; ${}^{1}$—CvsD (p=0.053); ${}^{2}$—BvsD (p=0.02).

**Table 5:** Comparison of the frequency of polymorphisms in the DRD2 receptor gene in subpopulations of patients according to the severity of alcohol craving (according to the PACS results) and the control group.

	Study			Control
	Participants			Group
	**A**	**B**	**C**	**D**
Gene	Patients	Patients	Patients	Subjects
polymorphisms	with PACS	with PACS	with PACS	with PACS
DRD2 (rs1800497)	0–3 points	4–9 points	>10 points	0–3 points
	N=26	N=44	N=35	N=50
No polymorphisms G/G	18 (69.2%)	25 (56.9%)	21 (60.0%)	28 (56.0%)
Polymorphism—heterozygote G/A	4 (15.4%) ${}^{1}$	17 (38.6%)	14 (40.0%) ${}^{2}$	20 (40.0%) ${}^{3}$
Polymorphism—mutational homozygote A/A (of clinical significance)	4 (15.4%)	2 (4.5%)	0 (0.0%) ${}^{2}$	2 (4.0%)

Pearson Chi-square with Yates’ correction; ${}^{1}$—AvsB (p=0.074); ${}^{2}$—AvsC (p=0.06–0.071); ${}^{3}$—AvsD (p=0.053).

**Table 6:** Comparison of the PACS score (severity of alcohol craving) according to polymorphisms in the COMT or DRD2 receptor gene in subpopulations of patients with alcohol dependence.

Variables	PACS x ± SD	*p*
COMT (rs4680)		
No polymorphisms G/G, n=26	8.27±6.10	
Polymorphism—heterozygote G/A, n=57	6.70±5.06	0.476
Polymorphism—mutational homozygote A/A (of clinical significance), n=21	8.38±6.59	
DRD2 (rs1800497)		
No polymorphisms G/G, n=63	7.16±5.74	
Polymorphism—heterozygote G/A, n=35	8.71±5.41	0.028
Polymorphism—mutational homozygote A/A (of clinical significance), n=6	2.83±3.81	

**Table 7:** Clinical characteristics, temperamental and character traits of the sub-population of patients experiencing different levels of alcohol craving—part 1.

Variables	(A)	(B)	(C)	(D)	*p*
	Patients	Patients	Patients	Control Group	
	PACS	PACS	PACS	PACS	
	0–3	4–9	>10	0–3	
	N=26	N=44	N=35	N=50	
	Mean±SD	Mean±SD	Mean±SD	Mean±SD	
Age (years)	38.8±8.5	39.0±8.4	36.5±5.9	38.32±9.86	0.658
Length of dependence	12.23±8.30	13.05±7.44	11.88±5.48	$0.00\pm 0.00{\;}^{3,4,5}$	0.001
Standard monthly number of drinks	173.69±251.34	295.10±372.69	304.86±210.52	$3.96\pm 11.85{\;}^{3,4,5}$	0.001
Longest monthly abstinence (days)	15.99±11.25	14.08±12.12	12.51±11.68	—	0.357
Longest abstinence last year (days)	80.75±95.65	58.89±74.82	58.62±65.53	$170.28\pm 127.18{\;}^{3,4,5}$	0.001
SADD	22.76±7.57	23.93±7.18	24.14±6.30	$0.58\pm 1.14{\;}^{3,4,5}$	0.001
Pre-meal hunger	12.08±7.31	13.59±6.54	13.94±5.84	$9.88\pm 7.68{\;}^{5}$	0.003
Antropometry					
Weight (kg)	76.90±10.90	77.60±16.33	80.81±18.18	83.57±19.91	0.459
BMI	24.95±3.28	24.93±4.20	26.29±4.91	25.88±4.86	0.359
% FM	19.12±6.29	19.80±6.78	21.72±7.12	$24.20\pm 7.35{\;}^{3,4}$	0.002
Phase angle	6.14±0.80	5.77±0.81	6.15±0.74	6.03±0.62	0.124
TCI: Temperamental Traits					
d—related to dopamine, s—related to serotonin, n—related to noradrenaline					
Search for novelty (d)	21.96±4.12	22.32±4.43	21.88±4.38	$17.49\pm 5.59{\;}^{3,4,5}$	0.001
Curiosity	5.92±1.70	5.32±2.10	5.06±2.15	5.69±2.60	0.315
Impulsiveness	4.84±1.84	5.03±1.73	4.19±1.59	$3.77\pm 1.79{\;}^{4}$	0.017
Extravagance	6.96±1.85	6.79±1.96	7.34±1.94	$4.28\pm 2.12{\;}^{3,4,5}$	0.001
Disorder	4.24±1.76	5.18±1.70	5.28±1.87	$3.74\pm 1.71{\;}^{4,5}$	0.001
Avoiding harm (s)	17.32±6.68	18.21±5.94	19.13±6.94	$14.64\pm 6.87{\;}^{5}$	0.032
Pessimism	4.60±2.44	5.03±2.06	5.78±2.22	$3.74\pm 2.37{\;}^{5}$	0.002
Insecurity	3.69±1.90	4.66±1.93	4.69±2.17	4.23±2.08	0.368
Social anxiety	4.08±2.32	4.16±2.13	4.06±2.18	3.69±2.07	0.769
Fatigue and asthenia	4.68±2.39	4.82±3.01	4.59±2.12	$2.97\pm 1.95{\;}^{3,4,5}$	0.001
Award dependence (n)	$15.64\pm 2.98{\;}^{1}$	13.47±3.19	14.91±3.67	14.15±3.71	0.106
Sentimentality	7.44±2.12	6.84±2.03	7.03±2.13	$5.82\pm 2.15{\;}^{3}$	0.016
Affection	4.76±1.53	$3.82\pm 1.54{\;}^{2}$	4.84±1.81	$4.92\pm 1.76{\;}^{4}$	0.019
Dependency	3.44±1.22	2.92±1.40	3.06±1.24	3.44±1.51	0.289
Perseverance	$4.76\pm 1.76{\;}^{1}$	3.47±2.14	4.06±1.84	4.54±1.73	0.045

Kruskal-Wallis ANOVA test; ${}^{1}$–AvsB, ${}^{2}$–BvsC, ${}^{3}$–AvsD, ${}^{4}$–BvsD, ${}^{5}$–CvsD; statistical significance p<0.05.

**Table 8:** Clinical characteristics, temperamental and character traits of the sub-population of patients experiencing different levels of alcohol craving—part 2.

Variables	(A)	(B)	(C)	(D)	*p*
	Patients	Patients	Patients	Control Group	
	PACS	PACS	PACS	PACS	
	0–3	4–9	>10	0–3	
	N=26	N=44	N=35	N=50	
	Mean±SD	Mean±SD	Mean±SD	Mean±SD	
TCI: Character Traits					
Self-Direction	$27.00\pm 8.07{\;}^{1}$	21.61±5.49	$21.09\pm 7.27{\;}^{2}$	$30.51\pm 7.70{\;}^{3,4}$	0.001
Responsibility	4.56±2.21	3.84±1.80	3.88±2.22	$6.10\pm 2.26{\;}^{2,3,4}$	0.001
Desirability of proceeding	5.48±1.41	4.55±1.37	4.53±2.03	$5.87\pm 1.68{\;}^{3,4}$	0.001
Resourcefulness	3.04±1.51	2.24±1.19	2.44±1.74	$3.36\pm 1.47{\;}^{3}$	0.003
Self-acceptance	$7.24\pm 2.48{\;}^{1}$	5.00±2.80	$4.72\pm 2.12{\;}^{2}$	$6.87\pm 2.35{\;}^{3,4}$	0.001
Second nature	6.68±2.56	5.97±1.71	5.53±2.17	$8.31\pm 2.61{\;}^{3,4}$	0.001
Willingness to cooperate	$32.28\pm 5.12{\;}^{1}$	28.18±6.54	29.16±6.02	$32.41\pm 5.87{\;}^{4}$	0.002
Social acceptance/tolerance	7.16±1.21	6.55±1.46	6.34±1.57	6.90±1.42	0.104
Empathy	$5.00\pm 1.35{\;}^{1}$	3.84±1.49	4.59±1.60	4.62±1.49	0.031
Readiness to help	$5.64\pm 1.28{\;}^{1}$	4.82±1.27	5.28±1.46	$6.10\pm 1.39{\;}^{3}$	0.001
Indulgence	7.76±2.33	6.84±2.77	6.78±2.68	7.77±2.41	0.228
Integrated conscience	6.72±1.20	6.13±1.69	6.16±1.74	7.03±1.47	0.042
Depersonalization	15.88±5.45	14.92±6.00	15.72±7.23	$11.44\pm 5.94{\;}^{2,4}$	0.010
Self-transcendence	5.56±1.91	6.11±2.25	5.97±2.63	$4.23\pm 1.98{\;}^{2,3,4}$	0.001
Transpersonal identification	4.72±2.11	3.68±2.36	4.38±2.67	$2.74\pm 2.11{\;}^{2,4}$	0.006
Spirituality	5.60±2.84	5.13±2.67	5.38±2.86	4.46±2.77	0.328

Kruskal-Wallis ANOVA test; ${}^{1}$—AvsB, ${}^{2}$—AvsD, ${}^{3}$—BvsD, ${}^{4}$—CvsD; statistical significance p<0.05.

**Table 9:** Regression of method backward elimination in the patients group with alcohol dependence.

Variables	Patients Alcohol Depend. Treat. Unit n= 94–105			
Dependence variables	Independent variables	Standardized Coefficient Beta	*p*	$R^{2}$
	TCI: Temperamental Traits	−0.173	0.077	
	Perseverance			0.211
PACS (score)	TCI: Character Traits	−0.411		
	Self-acceptance		<0.001	
	Polymorphism DRD2	−0.183	0.055	

## Data Availability

The data is available upon request from corresponding authors.
